# Care cascades for hypertension and diabetes: Cross-sectional evaluation of rural districts in Tanzania

**DOI:** 10.1371/journal.pmed.1004140

**Published:** 2022-12-05

**Authors:** Brianna Osetinsky, Grace Mhalu, Sally Mtenga, Fabrizio Tediosi

**Affiliations:** 1 Swiss Tropical and Public Health Institute, Allschwill, Switzerland; 2 University of Basel, Basel, Switzerland; 3 Ifakara Health Institute, Dar es Salaam, Tanzania

## Abstract

**Background:**

Noncommunicable diseases (NCDs), especially hypertension and diabetes, are rapidly rising in sub-Saharan Africa, necessitating health systems transformations. In Tanzania, current policies aim to improve control of hypertension and diabetes, but information is still needed to assess the gaps in treatment.

**Methods and findings:**

We conducted a cross-sectional household survey of 784 adults in two districts in Tanzania from December 2020 to January 2021, capturing the cascade-of-care for hypertension and diabetes. The ages of the respondents ranged from 18 to 89 years. Of those screened positive for these conditions, we measured the proportion in each step of the cascades: awareness, care engagement, treatment, and control. We conducted multivariable logistic regression analyses for all four steps along the hypertension care cascade with the independent variables of social health protection schemes, and prior diagnosis of comorbid diabetes, and demographic information. In our sample, of the 771 who had their blood pressure measured, 41% (95% confidence interval (CI): 38% to 44%) were screened positive for hypertension, and of the 707 who had their blood sugar measured, 6% (95% CI: 4% to 8%) were screened positive for diabetes. Of those with hypertension, 43% (95% CI: 38% to 49%) had a prior diagnosis, 25% (95% CI: 21% to 31%) were engaged in care, 21% (95% CI: 3% to 25%) were on treatment, and 11% (95% CI: 8% to 15%) were controlled. Of the 42 respondents with diabetes, 80% (95% CI: 69% to 93%) had a prior diagnosis. The diabetes care cascade had much less drop-off, so 66% of those with diabetes (95% CI: 52% to 82%) were engaged in care and on treatment, and 48% (95% CI: 32% to 63%) had their diabetes controlled at the point of testing. Healthcare fee exemptions were independently associated with higher odds of being previously diagnosed (OR 5.81; 95% CI [1.98 to 17.10] *p* < 0.005), engaged in care (OR 4.71; 95% CI [1.59 to 13.90] *p* 0.005), and retained in treatment (OR 2.93; 95% CI [1.03 to 8.35] *p* < 0.05). Prior diagnosis of comorbid diabetes was highly associated with higher odds of being engaged in care for hypertension (OR 3.26; 95% CI [1.39 to 7.63] *p* < 0.005). The two primary limitations of this study were reliance on screening at a single time point only of people available at the village at the time of the sample and dependence on self-report for to inform the three cascade steps of prior diagnosis, healthcare visits for engagement in care, and treatment use.

**Conclusions:**

The high burden of hypertension and low levels of control in our study underscores the importance of improving the awareness and treatment of hypertension. The differences in the care cascades for hypertension and diabetes demonstrates that chronic NCD treatment is possible in this setting, but efforts will be needed across the entire care cascade to improve hypertension control.

## Abstract 2

Dr. Brianna Osetinsky and colleagues report a cross sectional evaluation of care cascades for the delivery of hypertension and diabetes care in rural districts of Tanzania.

## Author summary

**Why was this study done?:**

- The rising prevalence of noncommunicable diseases (NCDs), especially cardiovascular disease and diabetes, in sub-Saharan Africa (SSA) requires transformations in health systems.
- As effective chronic NCD treatment requires diagnosis and care continuity, a care cascade can be used to monitor the effective management of hypertension and diabetes.
- In Tanzania, recent studies have found low levels of diagnostic tools, training of healthcare providers, and availability of essential medicines for NCDs across all levels of the health system.

**What did the researchers do and find?:**

- This study used a cascade of care approach to quantify engagement across the continua of chronic care and examined the correlates of hypertension awareness, engagement in care, retention on treatment, and control.
- We sampled 784 people in two rural districts of Tanzania and found that of the 771 who had their blood pressure measured, 316 people had hypertension, and of the 707 who had their random blood sugar measured, 42 had diabetes.
- In addition to blood pressure measurements, the rest of the care cascade was informed by respondents’ reports of prior diagnosis of hypertension and diabetes, most recent visit to a health facility for hypertensive or diabetic care, and medications taken in the last 7 days.
- Only 11% of those with hypertension had their condition controlled, due to low levels of awareness, engagement in care, and access to treatments. Almost half of those with diabetes had their diabetes controlled at the point of testing, with 66% of them engaged in care and on treatment.
- Predictors of progress along the hypertension care cascade included older age, more education, and exemptions for healthcare fees.

**What do these findings mean?:**

- These study findings indicate major gaps in the health system capacity to provide care to people with hypertension and diabetes in Tanzania.
- The substantial drop-off at each step of the hypertension care cascade shows that approaches focused only on single health system barriers to hypertension control are not enough as full control would require interventions for increasing awareness, engagement in care, and retention in treatment.
- While the number of people with diabetes in our sample is small, the higher proportion of people with diabetes, compared to those with hypertension, in each level of the care cascade suggests that improving chronic care is possible through efforts to improve care seeking and adherence to care.

## Introduction

The prevalence of noncommunicable diseases (NCDs), especially cardiovascular disease and diabetes, is rising sharply in sub-Saharan Africa (SSA) where the burden has increased 67% since 1990 [1]. It is estimated that over 125 million adults have hypertension, and 24 million adults have diabetes as of 2021, resulting in 2 million premature deaths every year in SSA [2,3].

This rapid epidemiological transition in SSA requires transformations in health systems. There is a need to strengthen primary care and address the long-standing constraints of underfunding and focus on episodic care for acute conditions [1,4]. While diabetes and hypertension can be effectively controlled with treatment, many health systems are currently underprepared to provide this care [5,6]. The persistently low levels of awareness and treatment for these conditions throughout SSA results in low levels of control, particularly in rural areas [7,8].

As effective chronic NCD treatment requires diagnosis and care continuity and coordination, a care cascade can be used to monitor the effective management of hypertension and diabetes [9]. The cascade-of-care, originally developed for chronic communicable diseases like HIV and tuberculosis, is a relevant framework for evaluating the health systems effectiveness for hypertension and diabetes care [10–12]. This method defines the proportion of people with a specific disease across steps of treatment, from screening to control. A cascade-of-care provides both a measure of disease control and identifies steps along the cascade with high proportion of loss to guide development of interventions and future policies.

In Tanzania, the Strategic and Action Plan for the Prevention and Control of NCDs was developed in 2016 to strengthen the health system capacity to provide appropriate chronic disease care [13]. However, current findings on the readiness to provide care have found low levels of diagnostic tools, training of healthcare providers, and availability of essential medicines for NCDs across all levels of the health system [14]. There are very low levels of hypertension and diabetes awareness and control across the country, with lower awareness in rural areas [15]. Poor adherence to care for those who have been diagnosed is driven by a combination of challenges including long waiting times, and poor point of care communication and patient understanding of the disease, availability of medication, and also high costs of care with regular visits, especially medication costs [16,17].

To identify the unmet needs in the care continuum for hypertension and diabetes, we have reported the prevalence of these conditions and used a cascade of care approach to quantify continued engagement across the continua of chronic care and examined the correlates of hypertension awareness, engagement in care, retention on treatment, and control. Our study aims to provide further insight into the barriers to control of hypertension and diabetes by incorporating three novel components along the steps of the care cascade. First, social health protection, such as health insurance, or waiving of healthcare fees, can address issues of direct healthcare costs. Separating the different insurance types and fee exemptions provides additional insight as coverage for medications differ. Relatedly, because of challenges in access to medicines both due to costs and stockouts, we differentiate between patients who are seeking care for their hypertension and diabetes, which is reflected in the care cascade step “engaged in care,” and those who are both engaged in care and on treatment, which includes accessing medicines if needed. Lastly, as we measured diabetes and hypertension in the same population, we can examine the differences in progression along the cascades of these two conditions, and the association between comorbid diabetes along the hypertensive care cascade. This is an important comparison because despite similar requirements of chronic care for both hypertension and diabetes, hypertension symptoms are less likely to be noticed by patients, which might impede patient demand for care or adherence to treatment, especially in the long term.

## Methods

### Study setting

Our study took place in the Kilombero district in the Morogoro region, and Same district in the Kilimanjaro region. Both districts are primarily rural with a town center at Ifakara and Same town, respectively. While these towns have populations under 100,000, town residents and those in peri-urban areas outside the town have closer access to the highest levels of care due to the hierarchical structure of the health system [18]. Village-level dispensaries provide basic care and are generally staffed with a nurse as the highest-level providers who treat lower complexity conditions and are able to prescribe medications for a limited range of treatments. This does not often include hypertension and diabetes. The next level is health centers, which are staffed by mid-level providers including clinical officers, nurses, and midwives, and can therefore provide care for more complex conditions and dispense for a wider range of treatments. Health centers are increasingly providing care for hypertension and diabetes on designated NCD days when a medical officer trained in this care is available, though they may also provide hypertension and diabetes care on other days. The next level is the district hospital, which provides both inpatient and outpatient services and are staffed by medical doctors, including some specialists and others who can provide hypertension and diabetes care for both straightforward and complex cases. The hospitals also provide NCD treatment days for hypertension and diabetes, similar to the health centers. The structure is intended to allow for patients with more complex conditions to be referred up the hierarchy, while primary care can be delivered at the local level, but the referral pathway is unenforced. The Same District hospital is located in Same Town, and St. Francis Referral Hospital is located in Ifakara.

Out-of-pocket payments to access health services are still very common, but efforts to increase social health protection to improve access to healthcare are underway in Tanzania. The improved Community Health Fund (iCHF) is a public insurance option aimed at the informal sector and provides coverage for care and medications at public health centers [19]. However, in the event of a stockout in public pharmacies, medicines are purchased in the private sector paying out of pocket. The National Health Insurance Fund (NHIF) provides comprehensive coverage including in private pharmacies but generally covers the formal sector and is mandatory enrollment within the civil service [20]. Healthcare fee exemptions are waivers granted for some elderly people, those with very low income, or for disease-specific healthcare needs such as HIV care. These waive all costs of care and medications at public facilities but do not require a premium payment to be enrolled like the health insurance programs do.

### Sampling and data collection

We conducted a population household survey from November 2020 to January 2021 using multistage cluster randomized sampling as part of a larger project investigating health expenditures and use of chronic disease care in Tanzania. The cascade-of-care analysis from the household surveys is included in the prospective protocol submitted for IRB (see S1 Protocol). Sample size was calculated based on a hypertension prevalence of 19.9% (95% confidence interval (CI): 17.1 to 22.9) and diabetes prevalence of 14.8% (95% CI: 12.4 to 17.6) for rural Tanzania adults, population estimates for Kilombero and Same districts from the Tanzania 2012 census, and a benchmark mean annual household health expenditure of 30,000 Tanzanian shillings [21]. Using a modified Cochran sample size calculation (power of 0.80, significance level of 0.05), we found a minimum sample size of 202 households per district is required. However, due to the limited information on the care cascades, we increased the total sample size to 390 households per district. We collected data in 26 village clusters per district, randomly sampling the cluster proportional to ward population size. As the Ifakara and Same Town Councils form their own administrative wards, street names were randomly selected as clusters, rather than village. Within each cluster 15 households were randomly selected. In the villages, households were selected by simple random sampling using a list of households provided by the village head. In Ifakara and Same Town, the randomly selected street names from the town councils served as the starting point for sampling, and each third household counterclockwise of the block was sampled. Village leaders (village executive officer or hamlet leader) assisted to introduce the research team to the households. The field worker explained the purpose of the visit was to ask about healthcare use and health expenditures in the household. She/he requested the consent to participate in the survey to one household representative over the age of 18 who would be available at some point during the day of data collection in the village either the head of household or a deputy who would be able to answer questions on household expenditures. To reduce the possibility bias if people concerned about their hypertension or diabetes status due to their age or symptoms would request to be the respondent, the focus on the explanation to the household head was on information about healthcare utilization. Furthermore, anyone in the household who was interested in being screened for hypertension and diabetes could be tested without inclusion in the sample. The field worker gave the respondents the option to have the written informed consent read to them, and those that could not write signed their assent with a fingerprint stamp.

The survey included demographic and socioeconomic questions and questions about healthcare utilization, as well as measurements of blood pressure and random blood sugar. Healthcare utilization questions included prior diagnosis of a chronic condition, past healthcare visits, and their current medications. Blood pressure was measured using a validated and calibrated digital blood pressure monitor (OMRON M6 Comfort HEM-7321-E). Respondents were seated and blood pressure was measured 3 times at least 5 minutes apart following WHO guidelines on the screening of blood pressure. The average of the second and third blood pressure measurements were used to determine blood pressure. We measured random blood glucose (RBG) using a point-of-care glucometer (On-Call Plus EZ II) and a blood draw from the second or third finger. If respondents had blood sugar or blood pressure above normal cutoffs, they were advised that their readings were elevated and they should follow up for further testing with a healthcare provider.

### Definition of the care cascade

A care cascade is defined as the share of people with a health condition along different steps of a treatment cascade. The cascades are restricted to patients who screened positive for hypertension or diabetes. Respondents were classified as having screened positive for hypertension if they have a prior diagnosis of hypertension and are in treatment, or if their mean systolic blood pressure (SBP) ≥140 mm Hg and/or mean diastolic blood pressure (DBP) ≥90 mm Hg, using the hypertension definition in use by the Republic of Tanzania and by WHO [22]. Respondents were classified as having diabetes if their RBG measurement was above 11 mmol/L or if they reported a diagnosis of diabetes and are in treatment with a RBG measurement below 11 mmol/L. Fasting status was assessed by asking time since last ate or drank any sweetened beverage, and for those who were fasted, we used the fasting blood glucose (FBG) cutoff of 7 mmol/L for diabetes. Those that reported a prior diagnosis and had normal biometric readings but were not on medication nor had they ever been engaged in care for the chronic disease they reportedly were diagnosed with were not considered to have the disease. This is to account for recall bias that might be caused by prior screening of elevated blood pressure or blood sugar, without an actual diagnosis, whitecoat syndrome for hypertension, resulting in prior high measurement that would have to have been confirmed with follow-up measurements, or general medical advice to be aware of blood pressure or blood sugar, since these people would not be accessing the health system for hypertension or diabetes and therefore should not be in the cascade. Some people have controlled hypertension or diabetes without medication, but since appropriate diagnosis requires several visits to a health facility, they would have been engaged in care at some point in the past, and they are still included as having the condition reported.

**Fig 1:**
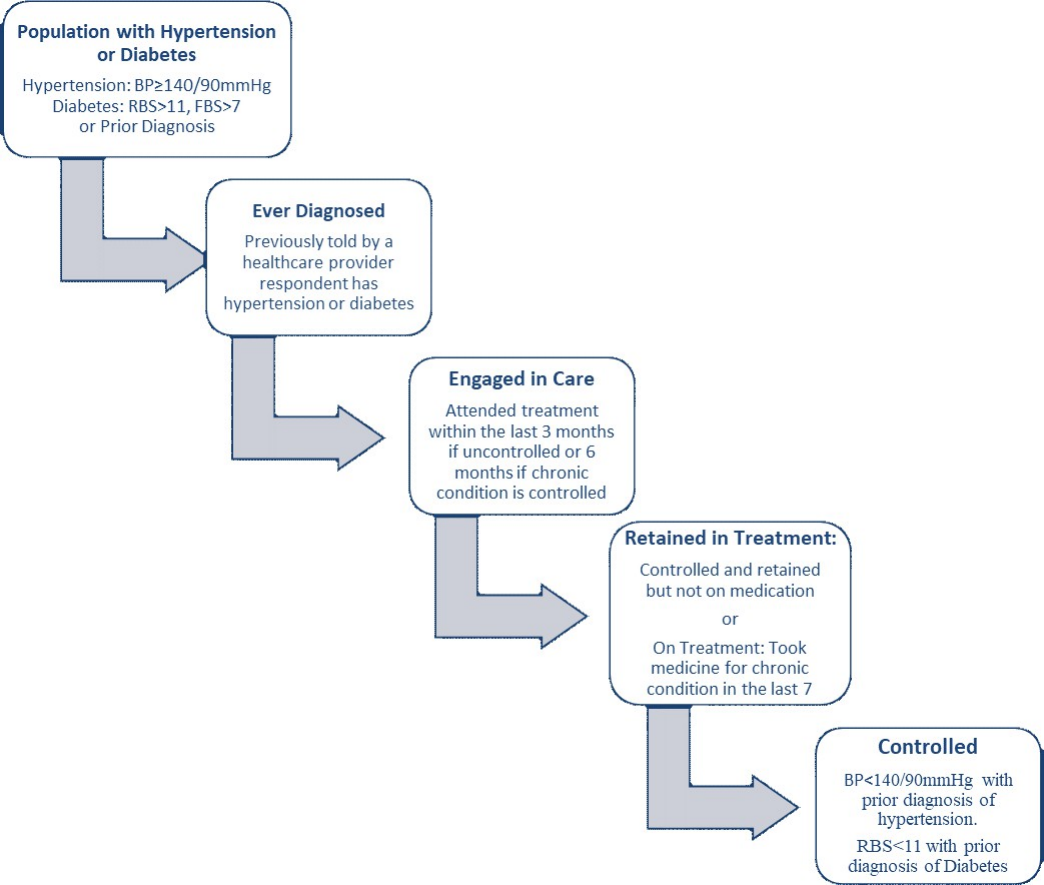
Cascade of care used for hypertension or dabetes. Progressing from the population with the chronic condition, to diagnosis, engagement in care, retained in treatment with either lifestyle or pharmaceutical treatments, and control of the chronic condition. BP, blood pressure; FBS, fasting blood sugar; RBS, random blood sugar.

We categorized four levels in the cascade of care for this analysis: diagnosed, engaged in care, retained in treatment, and controlled chronic condition (Fig 1).

Diagnosed respondents reported that a healthcare provider has diagnosed him or her with hypertension or diabetes.

Engaged in care respondents were previously diagnosed and they reported a healthcare visit for their chronic condition within the prior 3 to 6 months based on the Tanzanian standard treatment guidelines and practice for hypertension and diabetes, and the Tanzanian NCD Desk Guide [22,23]. Per the recommendation, people with uncontrolled hypertension should receive a healthcare visit every 3 months. Once hypertension is controlled with or without medication, it is recommended to visit a health center every 6 months. Therefore, we classified respondents as engaged in care for hypertension if those with a hypertension diagnosis reported having healthcare for their condition within the last 3 months, or within the last 6 months if their measured blood pressure was below 140/90 mm Hg. People with uncontrolled diabetes are recommended to have a healthcare visit every month. Those with controlled diabetes have a recommended follow-up schedule of every 3 months. Respondents with a diabetes diagnosis were considered engaged in care if they had reported accessing care for diabetes within the last month or within the prior 3 months if their RBG measured below 11 mmol/L in our survey.

Retained in treatment respondents were diagnosed, engaged in care, and reported taking prescribed medicine for hypertension or diabetes within the last 7 days. Because lifestyle modifications as directed by healthcare providers is also a treatment for both hypertension and diabetes, we also considered respondents as retained in treatment if they were engaged in care, and had not taken medication in the last 7 days for their condition, but had normal biometric measurements at the time of testing. For respondents with both hypertension and diabetes, they were considered retained in treatment for hypertension if the medications they reported taking included medications intended to treat hypertension, or they were engaged in care and not taking hypertension medicine but had normal blood pressure measurements at the time of testing.

Respondents had Controlled hypertension or diabetes if they were classified as having a prior diagnosis, and biometric measurements were within the normal range for their disease. As a chronic disease, throughout the life course individuals can conceivably move from diagnosed, to treated, retained, or controlled and back as their engagement with the health system changes. Our research only captures the point along the cascade at the time the individual completed the survey.

### Explanatory variables

Residential location was defined as rural or urban/peri-urban. Marital status was classified as married or living together, compared with those that were divorced, widowed, or single. Educational attainment was divided into three groups: did not complete primary school, primary school was the highest level of education completed, or Secondary School completed. Occupation was classified as formal sector employment through the civil service or in the private formal sector, farming, self-employed or employed at a small business, working for the home including caring for children, retired, and unemployed. Social health protection includes enrollment in either public health insurance option, iCHF or NHIF, enrollment in private health insurance, or receiving a healthcare fee exemption. Prior diagnosis of diabetes was for all respondents who reported that a healthcare provider had given them a diagnosis of diabetes or high blood sugar in the past.

### Data analysis

We evaluated the sociodemographic breakdown of our sample along our explanatory variables. We conducted bivariate analysis for each of the explanatory variables for the outcomes of screened positive for diabetes and screened positive for high blood pressure. We evaluated the prevalence of hypertension and diabetes, and proportion of those screened positive throughout the rest of the care cascade for each condition, both for the total sample and for subgroups by age, sex, and social health protection status. To test the hypothesis that social health protection was associated with engagement along the care cascade, we conducted multivariable logistic regression for each of the four stages of the hypertensive care cascade controlling for sociodemographic factors, and comorbid diabetes with prior diagnosis with clustered standard errors to account for the sampling strategy. We conducted sensitivity analysis on the definition of engagement in care (presented in S4 Table). We planned to include a separate multivariable logistic regression for the diabetes care cascade, however, as the sample size of those screened positive for diabetes was insufficient for this analysis. Data were cleaned and analyzed using Stata/IC 16. This study is reported as per the Strengthening the Reporting of Observational Studies in Epidemiology (STROBE) guideline (S1 Checklist).

### Ethics

Ethical clearance for this study was obtained from the Ifakara Health Institute (IHI) Institutional Review Board (IHI/IRB/EXT/No:35–2020) and the Tanzanian National Institute for Medical Research (NIMR/HQ/R.8a/Vol.IX/3518). While the screening of elevated blood pressure and blood sugar could not count as a diagnosis of hypertension or diabetes, we did provide counseling to those that screened as positive that their results indicated they needed medical care, and they were referred to their nearest health facility that provided hypertension and diabetes care. Additional information regarding the ethical, cultural, and scientific considerations specific to inclusivity in global research is included in the Supporting information (S2 Checklist).

## Results

Of the 784 people in the sample, 771 had complete blood pressure measurements, and 707 had agreed to test their blood sugar. In our sample, 316 of the 771 who had their blood pressure measured (41%) screened positive, and 42 of the 707 screened for diabetes (6%) screened positive for diabetes, and 32 of the 699 who were screened for both (4%) were classified as having comorbid hypertension and diabetes (Table 1). When the age and gender profile of the survey was adjusted to fit the underlying Tanzanian population, the prevalence of hypertension in this population was 24%, and the age-adjusted prevalence of diabetes was considerably lower than national estimates, at 2% (See S1 Table for population-adjusted estimates). Respondent who screened positive for diabetes were more likely to be from Kilombero district, older, retired, and enrolled in a social health protection program compared to those who were not screened positive. Those screened positive for hypertension were older, more likely to be retired, enrolled in a social health protection program, and have lower educational attainment than those who were not screened positive for hypertension. Of the 42 who screened positive for diabetes and had their blood pressure measured, 32 had comorbid hypertension (78%), while that reflected only 11% of the 317 who screened positive for hypertension.

**Table 1:** Baseline characteristics of study participants.

Characteristic	Total Sample	Screened Positive for Diabetes		Screened Positive for Hypertension	
	N/Mean (SD/%)	N/Mean (SD/%)	*P* Value^a^	N/Mean (SD/%)	*P* Value^a^
Respondents	784	707		771	
Screened Positive for Chronic Condition		42 (6%)		316 (41%)	
Kilombero District	392 (50%)	16 (38%)	<0.05	155 (49%)	0.7268
Same District	392 (50%)	26 (62%)		161 (51%)	
Male	273 (35%)	18 (43%)	0.1793	102 (32%)	0.248
Female	511 (65%)	24 (62%)		214 (68%)	
Age—years	47 (14.3)	61 (8.8)	<0.005	54 (12.8)	<0.005
Rural	544 (69%)	27 (64%)	0.507	212 (67%)	0.3271
Marital Status					
Married/Living Together	583 (82%)	28 (67%)	0.297	218 (69%)	<0.005
Divorced/Widowed/Single	201 (28%)	14 (33%)		98 (31%)	
Highest Level Education Completed					
Did Not Complete Primary School	150 (22%)	8 (19%)	0.6956	77 (24%)	<0.005
Primary School	522 (67%)	30 (71%)		213 (67%)	
Secondary School or Higher	112 (74%)	4 (10%)		26 (8%)	
Occupation					
Formal Sector (Civil Servant/Private Formal)	25 (4%)	1 (2%)	<0.05	7 (2%)	<0.005
Farming	558 (79%)	28 (67%)		227 (72%)	
Self-Employed/Small Business	139 (20%)	6 (14%)		50 (16%)	
Retired	31 (4%)	6 (14%)		22 (7%)	
Other including Care for Home or Children	30 (4%)	1 (2%)		10 (3%)	
Social Health Protection^b^					
No Social Health Protection	634 (81%)	27 (64%)	<0.05	238 (75%)	<0.005
iCHF Health Insurance^c^	33 (4%)	4 (10%)		13 (4%)	
NHIF Health Insurance^d^	74 (9%)	8 (19%)		36 (11%)	
Other Health Insurance^e^	11 (1%)	1 (2%)		8 (3%)	
Healthcare Fee Exemption	38 (5%)	2 (5%)		24 (8%)	
Screened Positive for Comorbid Hypertension and Diabetes	32 (4%)	32 (78%)^g^	<0.005	32 (11%)^f^	<0.005

^a^*P* value from χ^2^ to compare *t* tests of samples with and without diabetes, and with and without hypertension.

^b^Respondents can both be enrolled in health insurance and have a fee exemption, so does not sum to 784, as 6 respondents had both.

^c^iCHF is the improved Community Health Fund.

^d^NHIF is the National Health Insurance Fund.

^e^Other Health Insurance can be privately bought nonpublic health insurance options.

^f^Of all screened positive for diabetes.

^g^Of all screened positive for hypertension.

**Fig 2:**
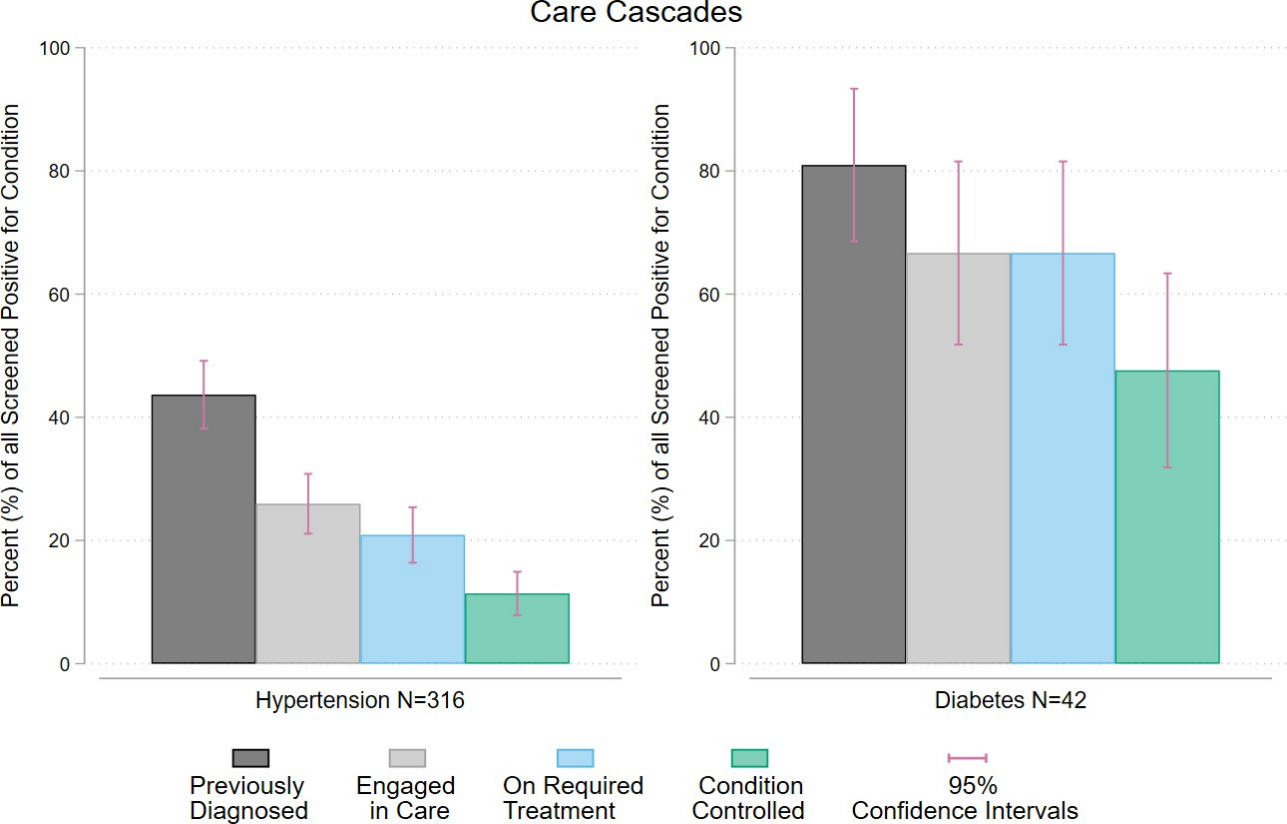
Care cascade for hypertension or diabetes, showing the percentage of all people who screened positive for the condition in each of the levels of the cascade: diagnosed, engaged in care, retained on treatment including lifestyle changes and pharmaceutical interventions, and controlled.

Fig 2 shows the percentage of those screened positive for hypertension or diabetes in each step in the care cascades. Of the 316 people screened positive for hypertension, 138 (43%, 95% CI: 38% to 49%) had no prior diagnosis of hypertension and 82 (25%, 95% CI 21% to 30%) were engaged in care. The next step, 66 of the 316 screened positive for hypertension were on treatment meaning they were on medication or retained in care and controlled without medicine (21%, 95% CI: 16% to 24%). At the far end of the cascade, 36 (11%, 95% CI: 8% to 15%) of those screened positive had their hypertension controlled at the point of testing. Of the 42 who screened positive for diabetes, 34 had prior diagnosis (81%, 95% CI: 69% to 93%) had no prior diabetes diagnosis while 28 (66%, 95% CI: 52% to 82%) engaged in care. All of those who were diagnosed and engaged in care were also retained in treatment. Of the 42 people screened positive for diabetes 50 (48%, 95% CI: 32% to 63%) had their diabetes controlled at the point of testing. The care cascades also differ across gender, age, and social health protection schemes, which can be used for further priority setting (see S1, S2, S3, and S4 Figs, demonstrating care cascade by these variables).

**Fig 3:**
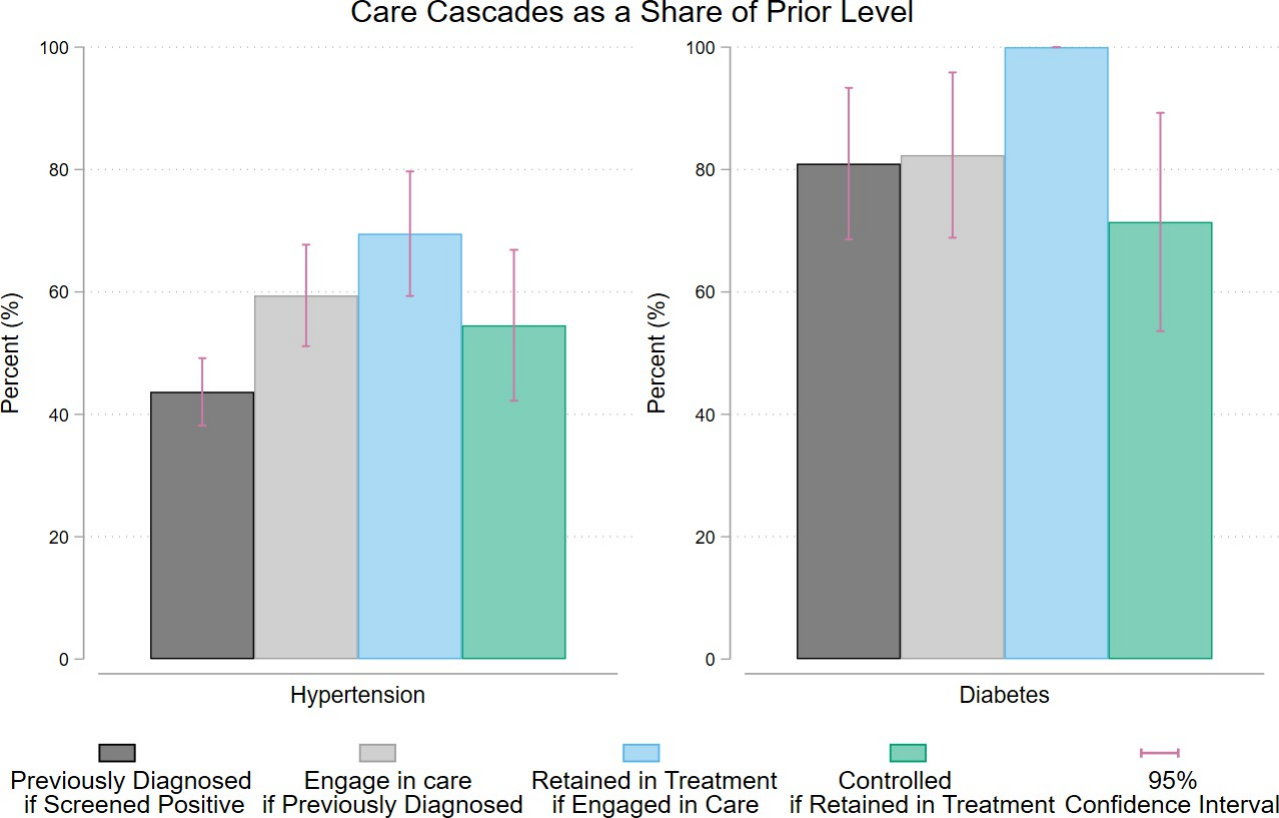
Steps in the care cascade by percent of prior level for hypertension and diabetes. This shows the share of people who were previously diagnosed with the condition if they screened positive, the percent who were engaged in care if previously diagnosed, the percent retained in treatment if they were engaged in care, and the percent that had their condition controlled if they were retained in treatment.

Fig 3 shows the mean prevalence in each step of the cascade as a share of the prior step. For people with hypertension, 45% (95% CI: 38% to 49%) had a previously been diagnosed with hypertension by a healthcare professional, 55% (95% CI: 47% to 64%) who had a prior diagnosis were engaged in care. Of those engaged in hypertensive care, 71% (95% CI: 61% to 81%) were actively retained in treatment, meaning they took medicine for their hypertension in the prior 7 days or they were controlled without medicine. Of those who were retained in hypertensive treatment, 43% (95% CI: 31% to 55%) were controlled. For those with diabetes, 86% (95% CI: 75% to 98%) had previously been told by a healthcare professional they had diabetes, 78% (95% CI: 63% to 93%) of people with a prior diagnosis were engaged in care, and 100% of those engaged in care were retained. Of the people engaged and retained in care, 69% (95% CI: 50% to 88%) had controlled diabetes at the point of testing.

Table 2 provides the odds ratios from the multivariable regression analysis for each of the four steps of the hypertension care cascade. Males had much lower odds of being previously diagnosed for hypertension (OR 0.29; 95% CI [0.15 to 0.56] *p* < 0.005) compared to females. Increasing age was associated with increased odds of being previously diagnosed for hypertension (OR 1.05; 95% CI [1.02 to 1.07] *p* < 0.005), engaged in care (OR 1.04; 95% CI [1.01 to 1.07] *p* < 0.05), and retained in treatment (OR 1.03; 95% CI [1.01 to 1.05] *p* < 0.05). Completing primary school was also associated with higher odds of being diagnosed, engaged in care, and retained in treatment compared to not completing primary school. People with hypertension who had a prior diagnosis of diabetes had considerably higher odds of being engaged in care (OR 3.26; 95% CI [1.39 to 7.63] *p* < 0.005). Controlling for the demographic characteristics included in Table 2, and the prior diagnosis of diabetes, the NHIF enrollment was associated with higher odds of retention in treatment (OR 3.18; 95% CI [1.08 to 9.36] *p* < 0.05). Respondents with healthcare fee exemptions had higher odds of being previously diagnosed (OR 5.81; 95% CI [1.98 to 17.10] *p* < 0.005), engaged in care (OR 4.71; 95% CI [1.59 to 13.90] *p* < 0.005), and retained in treatment (OR 2.93; 95% CI [1.03 to 8.35] *p* < 0.05). *P*s are derived from the Wald χ^2^ test.

**Table 2:** Multivariable regression analysis^†^ for each of the four steps of the Hypertension care cascade.

CHARACTERISTIC	PREVIOUSLY DIAGNOSED		ENGAGED IN CARE		RETAINED TREATED		CONTROLLED	
EVENTS IN STAGE	*N* = 138		*N* = 82		*N* = 66		*N* = 36	
	OR [95% CI]	*P* value	OR [95% CI]	*P* value	OR [95% CI]	*P* value	OR [95% CI]	*P* value
**KILOMBERO**	Reference Category		Reference Category		Reference Category		Reference Category	
**SAME**	1.02 [0.55,1.89]	0.985	1.20 [0.71,2.03]	0.486	1.55 [0.81,2.97]	0.187	1.92 [0.86,4.30]	0.111
**FEMALE**	Reference Category		Reference Category		Reference Category		Reference Category	
**MALE**	0.29 [0.15,0.56]	<0.005	0.51 [0.23,1.11]	0.089	0.48 [0.20,1.15]	0.101	0.46 [0.19,1.09]	0.077
**AGE**	1.05 [1.02,1.07]	<0.005	1.04 [1.01,1.07]	<0.05	1.03 [1.01,1.05]	<0.05	1.02 [0.98,1.05]	0.336
**URBAN/PERI-URBAN**	Reference Category		Reference Category		Reference Category		Reference Category	
**RURAL**	0.74 [0.39,1.40]	0.353	0.69 [0.39,1.23]	0.207	0.70 [0.36,1.35]	0.281	0.81 [0.40,1.64]	0.563
**MARITAL STATUS**								
** MARRIED/LIVING TOGETHER**	Reference Category		Reference Category		Reference Category		Reference Category	
**DIVORCED/** **WIDOWED/SINGLE**	0.49 [0.25,0.94]	<0.05	0.48 [0.25,0.93]	<0.05	0.50 [0.22,1.14]	0.097	0.52 [0.21,1.26]	0.146
**HIGHEST LEVEL EDUCATION COMPLETED**								
** DID NOT COMPLETE ANY SCHOOL**	Reference Category		Reference Category		Reference Category		Reference Category	
** PRIMARY SCHOOL**	1.91 [1.04,3.50]	<0.05	2.02 [0.98,4.18]	0.058	2.56 [1.01,6.53]	<0.05	1.93 [0.62,6.03]	0.257
** SECONDARY SCHOOL OR HIGHER**	3.27 [0.96,11.1]	0.058	2.17 [0.43,10.9]	0.348	2.74 [0.76,9.87]	0.124	2.85 [0.62,13.1]	0.179
**OCCUPATION**								
**FORMAL SECTOR** **(CIVIL SERVANT/PRIVATE FORMAL)**	Reference Category		Reference Category		Reference Category		Reference Category	
** FARMING**	2.16 [0.22,21.0]	0.506	0.98 [0.059,16.3]	0.987	1.12 [0.11,11.4]	0.927	0.45 [0.066,3.12]	0.420
**SELF-EMPLOYED/** **SMALL BUSINESS**	2.70 [0.24,30.5]	0.423	0.86 [0.043,17.2]	0.921	1.15 [0.10,13.1]	0.908	0.50 [0.074,3.34]	0.472
** CARE FOR HOME/CHILDREN**	2.51 [0.19,33.6]	0.488	2.61 [0.093,72.7]	0.572	3.31 [0.19,59.1]	0.416	1.24 [0.078,19.5]	0.880
** RETIRED**	12.2 [0.87,169.0]	0.063	3.22 [0.13,77.2]	0.470	1.40 [0.12,16.2]	0.786	0.38 [0.051,2.85]	0.348
**SOCIAL HEALTH PROTECTION**								
** NONE**	Reference Category		Reference Category		Reference Category		Reference Category	
**ICHF HEALTH INSURANCE**^**A**^	0.39 [0.057,2.64]	0.334	1.07 [0.21,5.36]	0.936	1.81 [0.42,7.79]	0.423	1.71 [0.24,12.1]	0.590
**NHIF HEALTH INSURANCE**^**B**^	1.87 [0.69,5.07]	0.217	2.00 [0.67,5.93]	0.214	3.18 [1.08,9.36]	0.036	1.35 [0.42,4.33]	0.618
** OTHER PRIVATE INSURANCE**	0.85 [0.14,5.25]	0.864	0.68 [0.12,3.87]	0.661	1.37 [0.25,7.34]	0.716	1.24 [0.15,9.99]	0.841
** HEALTH FEES EXEMPTION**	5.81 [1.98,17.1]	<0.005	4.71 [1.59,13.9]	<0.005	2.93 [1.03,8.35]	<0.05	2.48 [0.75,8.26]	0.138
**DIAGNOSIS OF COMORBID DIABETES**	2.32 [0.87,6.14]	0.091	3.26 [1.39,7.63]	<0.05	2.01 [0.88,4.62]	0.099	1.78 [0.64,4.96]	0.268

^†^This multiple variable analysis controls for all variables included in this table: district, gender, age, urban/rural, marital status, highest level of education completed, occupation, social health protection, and diagnosis of comorbid diabetes. For categorical variables, the reference category is the baseline against which the other categories are compared.

^a^iCHF is the improved Community Health Fund.

^b^NHIF is the National Health Insurance Fund.

## Discussion

The increasing burden of NCDs such as hypertension and diabetes is one of the major challenges for contemporary health systems especially in low-resource settings. This study shows that controlling hypertension and diabetes requires broad systemic approaches to strengthen both prevention and treatment especially in rural settings.

Narrow interventions focused only on single health system barriers to hypertension control are not enough, as indicated by the substantial drop-off at each step of the hypertension care cascade. As 59% of those screened hypertensive were unaware of their condition, screening efforts to improve awareness are potentially very important to improve control. However, screening efforts alone would be insufficient to improve health outcomes as other studies have found that linkage to care after a hypertension diagnosis is very low in Tanzania [24]. This is supported by our findings that 39% of those who were aware of their hypertension were not engaged in care, and 29% of those engaged in care were not on any treatment. The difference between engagement in care and on treatment provides a subtle but crucial distinction of people seeking care that are not accessing the treatments needed to control their condition, underscoring limitations in accessing medicines for hypertension. While other studies on antihypertensive drugs have reported high costs of medicines being a substantial barrier, 84% of the respondents in our survey who did not obtain all of their medications at the last health facility visit for their chronic condition reported that their prescribed medication was out of stock at the health facility (see S3 Table) [25]. This points to the importance of investments not only in medicines procurement but also in improving the efficiency of the whole medicines supply chain system. Additionally, even of those on treatment 57% are uncontrolled, meaning that less than 8% of people with hypertension have the condition under control.

Males had much lower odds of being previously diagnosed with hypertension, consistent with other research showing that men have lower engagement in the health system compared to females [26,27]. Older adults were more likely to be diagnosed, engaged in care, and retained in treatment, which may be related to perceptions of risk for cardiovascular events. While the 10-year cardiovascular disease risk is higher for older adults, younger people are still at risk for premature mortality. Risk also increases based on length of time that people are hypertensive without control, meaning that increasing diagnosis and treatment of people under 60 would further improve health outcomes. Despite the higher odds of treatment for older adults, they did not have higher levels of control, which is consistent with other research in SSA [28]. This might indicate poor quality of care received, especially regarding counseling, or need for additional caregiver support for older adults, as forgetfulness has been cited as a key barrier to medication adherence [25].

A higher level of education was also associated with higher odds of progressing along the care cascade, which might be associated with either higher levels of health literacy or higher socioeconomic status. We found no significant rural–urban differences in the care cascade. However, the urban wards were considerably smaller than many other cities in Tanzania, so the differences between lifestyle and proximity to healthcare facilities may be less extreme than other settings.

Low numbers of people with health insurance means it is hard to draw any conclusions regarding the associations between insurance on hypertensive care cascade (number of events for each covariate along the hypertensive care cascade is shown in S2 Table). NHIF coverage was associated with higher odds of being on treatment, but not associated with awareness, engagement in care, or control. One possible driver of this could be the comprehensive benefits of the NHIF including purchasing medications at private pharmacies. This would reduce the barrier of pharmacy stockouts and the higher prices of private pharmacies. Those with healthcare fee exemptions had higher odds of being diagnosed, engaged in care, and on treatment, suggesting that they work to improve access to care. Further research into the policies around healthcare fee exemptions, the population that receives them, and the other benefits associated with these exemptions would provide useful insight to direct policy changes to maximize the effect of these fee waivers. The respondents in our sample had higher levels of diagnosis, treatment, and control compared to other estimates of the hypertensive care cascades in SSA and were comparable to the mean level of diagnosis and control for other low- and middle-income countries (LMICs) [4]. However, this is still below the so-called “rule of halves” used to conceptualize the large proportion of people with undiagnosed, untreated, and uncontrolled hypertension, and very far below the WHO Global Action Plan for the Prevention and Control of NCDs target of 50% of eligible people receive drug therapy and counselling to prevent heart attacks and strokes [29,30]. Engagement in the care cascade for diabetes was much higher. Only 14% of those who screened positive for diabetes were unaware, and only 12% of those who had previously been diagnosed were not engaged in care. All of those engaged in care for diabetes were also on treatment, which is very different from those engaged in care for hypertension. Of those in care and on treatment, only 29% were uncontrolled, resulting in an overall control of diabetes of 54%. The differences of the care cascade for diabetes and hypertension may come from differences in patient demand, as hypertension is generally asymptomatic and recent health literacy campaigns in Tanzania have emphasized the threat of diabetes, which may result in more people seeking care and then adhering to treatment plans [31]. This possible driver is also suggested in research that found a common barrier in awareness and management of hypertension in Tanzania was the poor understanding of the chronicity of the diseases and associated long-term treatment [17]. The benefits of the increased engagement with the health system among people with a prior diabetes diagnosis in our sample also carried over to those with comorbid hypertension. When examining the multivariable analysis for the hypertensive care cascade, a diagnosis of comorbid diabetes is also strongly associated with higher odds of awareness, engagement, treatment, and control.

While the number that screened positive for diabetes is considerably smaller than for hypertension, the high levels of awareness, treatment, and control for diabetes suggests that improvements along the entire cascade are also possible for hypertensive treatment in this same setting with targeted interventions. Compared to other LMICs, especially in SSA, the respondents in our sample were more likely to have a prior diagnosis, be on treatment, and have control of their diabetes [32]. Our sample also had considerably lower prevalence of diabetes compared to other samples in Tanzania [21,32,33]. We relied primarily on a random blood glucose test as out of the 707 people who had their blood sugar tested, only 39 were in a fasted state. As a result, there may be people with diabetes in the population that were not included in this analysis, thereby biasing the sample to those with more extreme cases of diabetes or who were already in care. The low prevalence in our sample may be reflective of regional differences in prevalence. In a sample of three sites in eastern Africa, the prevalence varied significantly from 16% in rural Uganda to 8% in urban Tanzania [34]. Taking into account these limitations and the small sample of people with diabetes in our study, we must interpret the findings on diabetes care with caution.

This study has some other limitations that are important to consider in the interpretation of the results. We are reliant on patient self-report that a healthcare provider had given them a prior diagnosis of an NCD, and the timing of the most recent healthcare visits for a chronic condition, and current treatment use. Patient records from health facilities might have provided more detailed records of diagnosis or care engagement, but that would not be possible nor comprehensive in this setting. We also estimated the spacing of follow-up visits to classify engagement in care, rather than follow-up schedules tailored to individual patients. Our definition of hypertension and diabetes is also limited as it was measured at a single time point. While this is standard protocol for screening, actual diagnosis in health facilities relies on several separate measurements at different visits. Diabetes diagnosis, in particular, would generally require an additional fasting blood sugar measurement or hemoglobin A1C. Our reliance on sampling the self-identified household head who was available on the day the data collectors were in the town may bias the sample towards older respondents and may be the cause of the imbalance of the genders. Therefore, as this study was conducted in two districts in Tanzania that are mainly rural and overrepresent women and older people compared to the underlying population, the generalizability of the results is limited.

Despite these limitations, this study is, to our knowledge, the first study that measured the care cascade for both hypertension and diabetes among adults of all ages in Tanzania. The care cascades for hypertension and diabetes demonstrate differences in drop-off for each step. Understanding differences in care delivery, health education, and patient demand between the two conditions could help designing policies to improve awareness, engagement in care, retention in treatment, and control of hypertension. These policies will have to promote a systemic approach to strengthen the whole social health protection system to address all barriers to hypertension and diabetes control.

## Supporting information

S1 Checklist STROBE checklist. STROBE checklist for care cascades for hypertension and diabetes: Cross-sectional evaluation of rural districts in Tanzania.
(PDF)

S2 Checklist Inclusivity in global research checklist. (PDF)

S1 Protocol Prospective analysis plan as included in ethics approval. (DOCX)

S1 Table Population-adjusted prevalance and care cascade for hypertension and diabetes. (DOCX)

S2 Table Care cascade number of events in each stage of the care cascade. (DOCX)

S3 Table Reasons why medication was not obtained at the last healthcare visit. (DOCX)

S4 Table Sensitivity analysis, regression results for the outcome engagement in care varying definition of engaged in care. (DOCX)

S1 Fig Hypertensive care cascade divided by age groups and gender. This shows the proportion of those who screened positive for hypertension in the farthest point along the hypertension care cascade at the point of measurement, disaggregated by age group and gender.
(TIF)

S2 Fig Diabetic care cascade divided by age groups and gender. This shows the proportion of those who screened positive for diabetes in the farthest point along the diabetes care cascade at the point of measurement, disaggregated by age group and gender.
(TIF)

S3 Fig Hypertension care cascade by social health protection schemes. This shows the proportion of those who screened positive for hypertension in the farthest point along the hypertension care cascade at the point of measurement, disaggregated by social health protection status.
(TIF)

S4 Fig Diabetes care cascade by social health protection schemes. This shows the proportion of those who screened positive for diabetes in the farthest point along the diabetes care cascade at the point of measurement, disaggregated by social health protection status.
(TIF)

## Abbreviations

CI: confidence interval
DBP: diastolic blood pressure
FBG: fasting blood glucose
iCHF: improved Community Health Fund
IHI: Ifakara Health Institute
LMIC: low- and middle-income country
NCD: noncommunicable disease
NHIF: National Health Insurance Fund
RBG: random blood glucose
SBP: systolic blood pressure
SSA: sub-Saharan Africa
