# Supplementary material for: Care cascades for hypertension and diabetes: Cross-sectional evaluation of rural districts in Tanzania
Source: PLoS Med. 2022 Dec 5;19(12):e1004140. doi: 10.1371/journal.pmed.1004140 (PMC9762578; doi:10.1371/journal.pmed.1004140)
Supplement: S1 Protocol — (DOCX) [file pmed.1004140.s003.docx]

# Research for development (R4D): Health systems governance for an inclusive and sustainable social health protection in Ghana and Tanzania - Phase 2; Sub Study: Implementation of health financing policy and social health protection schemes in Tanzania

## Executive Summary

The Ifakara Health Institute, in collaboration with the Swiss Tropical and Public Health Institute, is seeking ethical clearance for the below protocol investigating the accessibility of NCD care in Tanzania, as a sub-study in the second phase of the Research for Development (R4D): Health systems governance for an inclusive and sustainable social health protection in Ghana and Tanzaia. Chronic non-communicable diseases (NCD), especially cardio-metabolic disorders such as hypertension and diabetes, are on the rise in sub-Saharan Africa. However, health systems in sub-Saharan Africa are mainly equipped for acute episodic treatment, and as such are ill-equipped for the successful treatment of non-communicable, chronic conditions. As such, the objectives of this protocol are to explore the implementation of health financing strategies particularly related to geographic accessibility of NCD care, economic barriers, willingness to pay for access to chronic disease care, and engagement in chronic care. This protocol will utilise both secondary and primary data to meet these objectives, and will begin by analysing secondary data provided by the National Health Insurance fund in order to provide an overview of NCD care provision in Tanzania and build a geospatial model of NCD care accessibility across Tanzania. Primary data will then be collected by recruiting households and patients to participate in surveys in order to better understand the costs of accessing NCD care, predictors of disengagement from care, and the willingness-to-pay for NCD care. Quantifying patient access and utilization of NCD care in Tanzania will provide crucial evidence for health systems strengthening efforts to provide NCD care in Tanzania, the importance of which will only increase as the burden of NCDs continues to rise due to demographic changes and urbanisation. In doing so, this study seeks to improve the equity of access to chronic care and inform the improvement of health financing policy and implementation so that Tanzania may work towards achieving a more inclusive, sustainable, and equitable health system and social health protection system.

# Table of Contents

[Research for development (R4D): Health systems governance for an inclusive and sustainable social health protection in Ghana and Tanzania - Phase 2; Sub Study: Implementation of health financing policy and social health protection schemes in Tanzania 0](#_Toc119682884)

[Executive Summary 0](#_Toc119682885)

[Table of Contents 1](#_Toc119682886)

[Abbreviations 2](#_Toc119682887)

[Introduction and Literature Review 3](#_Toc119682888)

[Statement of Problem, and Rationale 3](#_Toc119682889)

[Objectives and Research Aims 4](#_Toc119682890)

[Research Question 1 4](#_Toc119682891)

[Research Question 2 5](#_Toc119682892)

[Research Plan and Methods 5](#_Toc119682893)

[Primary Data Collection and Analysis 5](#_Toc119682894)

[Study Sites 5](#_Toc119682895)

[Study Design 5](#_Toc119682896)

[Sampling Strategy and Sample Size 5](#_Toc119682897)

[*Household survey* 5](#_Toc119682898)

[*Exit survey* 6](#_Toc119682899)

[Data Management 8](#_Toc119682900)

[Analytical Methods 8](#_Toc119682901)

[Research Ethics 9](#_Toc119682902)

[Dissemination Plan 10](#_Toc119682903)

[List of Investigators and their Roles 10](#_Toc119682904)

[References 10](#_Toc119682905)

[Annex I 12](#_Toc119682906)

[Tables of requested variables NHIF 12](#_Toc119682907)

# Abbreviations

| COSTECH | Tanzania Commission for Science and Technology |
| --- | --- |
| DHIS2 | District Health Information System 2 |
| EKNZ | Ethikkommission Nordwest- und Zentralschweiz |
| iCHF | Improved Community Health Financing |
| iHEA | International Health Economics Associated |
| IHI | Ifakara Health Institute |
| IRB | Institutional Review Board |
| MoHCDGEC | Ministry of Health, Community Development, Gender, Elderly and Children |
| NCD | Non-Communicable disease |
| NHIF | National Health Insurance Fund |
| NIMR | Tanzanian National Institute for Medical |
| OOP | Out of Pocket expenditures |
| PoRALG | President’s Office, Regional Administration and Local Government |
| R4D | Research For Development |
| SDC | Swiss Agency for Development and Cooperation |
| SNF | Swiss National Science Foundation |
| Swiss TPH | Swiss Tropical and Public Health Institute |
| WHO | World Health Organization |

# Introduction and Literature Review

Chronic non-communicable diseases (NCD), especially cardio-metabolic disorders such as hypertension and diabetes, are on the rise in sub-Saharan Africa. However, health systems in sub-Saharan Africa are mainly equipped for acute episodic treatment, and as such are ill-equipped for the successful treatment of chronic conditions [1]. This rapid epidemiological transition in the contribution of NCDs to the burden of disease in Africa has made it difficult for health systems to adapt to the challenge of financing and delivering costly chronic care. In Tanzania, several studies have evaluated the readiness and capacity of the health system to deliver chronic care, and have found very limited capacity in rural and primary care facilities in particular [2,3]. The lack of funding, and the inequitable allocation of funding for NCDs has exacerbated human resource challenges, and contributed to the poor availability of NCD diagnosis and care services outside major towns and urban centres [4]. The poor availability of NCD care at the primary care level has contributed to poorer health outcomes for Africans with NCDs, with age-standardised mortality attributable to NCDs being up to three times higher among sub-Saharan African populations, than among European ones. Therefore, these issues justify an in-depth investigation into the accessibility of NCD care in Tanzania, with the objective of identifying key barriers to NCD care accessibility and delivery, and making policy recommendations for strengthening and improving the delivery of NCD services in Tanzania.

# Statement of Problem, and Rationale

Quality NCD care is frequently unavailable at health facilities serving non-urban areas [2,3], 2014), and only approximately 32% of the population is covered by social health protection [5]. These issues increase the cost and difficulty of regularly accessing NCD care, so quantifying patient access and utilization of NCD care in Tanzania will provide crucial evidence for health systems strengthening efforts to provide chronic care.

National health Insurance Fund (NHIF) claims would provide a comprehensive record of healthcare received for NHIF beneficiaries, which can provide insight into the breadth and availability of NCD and other healthcare services that are available in Tanzania. While the NHIF only insures approximately 7% of the population [6], its comprehensive benefits package and nation-wide coverage jurisdiction make its claims database ideal for assessing healthcare provision, and otherwise costly chronic NCDs in particular [7]. Household surveys and health facility exit interviews will allow the in-depth investigation of health care utilisation, and the associated costs and structural challenges to using social health protection schemes to access care as reported by patients.

The current research proposal (R4D phase 2 Sub-study) will specifically explore the implementation of health financing strategies particularly related to geographic accessibility of NCD care, economic barriers, willingness to pay for access to chronic disease care, and engagement in chronic care. In doing so, this study seeks to improve the equity of access to chronic care and inform the improvement of health financing policy so that Tanzania may work towards achieving more equitable financing schemes inclusive of vulnerable and marginalized population groups.

# Objectives and Research Aims

Given the importance of health financing for access to and appropriate utilization of care, especially for chronic conditions, we are submitting a sub-study of the previously accepted protocol, “Health systems governance for an inclusive and sustainable social health protection in Tanzania.” The overall aim of our study is to explore the implementation process of health financing policy and social health protection schemes in Tanzania. In doing so, this study seeks to improve the equity of access to chronic care and inform the improvement of health financing policy so that Tanzania may work towards achieving more equitable financing schemes inclusive of vulnerable and marginalized population groups. In this phase of the study, we will expand upon the specific study objective *to identify challenges hindering the existing social health protection schemes from enabling poor and vulnerable groups accessing quality health care* with four specific objectives:

1. to quantify the direct and indirect cost associated with accessing NCD care services, and describe strategies for coping with the cost burden
2. to understand the differences between people with NCDs who are engaged in care and those that are not

To address these aims we will investigate the following research questions.

### Research Question 1

The second research question will aim to quantify the direct and indirect cost associated with accessing NCD care services, and describe strategies for coping with the cost burden. This includes determining which support systems people living with NCDs utilise, including their social networks, to cope with these costs. It will also seek to determine the cost of not having social health protection by comparing the total costs incurred between NHIF beneficiaries, iCHF beneficiaries, and those who are not part of a social health protection scheme. The role of patient social networks will be especially important to investigate, as it has been reported that people often attempt to capitalise on their social networks to avoid out-of-pocket expenditure (OOPs) by being added to another household’s iCHF membership, or by borrowing money from their social network [8]. Furthermore, this objective will take into account that although beneficiaries of social health protection may be less likely to experience financial hardship due to the direct cost of their illness, their illness may still impose a social and economic burden in the form of missed work days and lost income for either the individual living with the NCD, or their caregiver at times when one’s illness prevents them from working [9].

### Research Question 2

The third research question aims to understand the differences between people with NCDs who are engaged in care and those that are not. We aim to identify the proportion of the population that remains undiagnosed, and undertreated for hypertension and diabetes, and the barriers or perceived barriers to appropriately utilising NCD treatment compared with the patients who are accessing NCD care. In addition to socio-demographic and geographic mediators of care utilisation, we will quantify the willingness-to-pay for chronic disease care under different payment options, specifically out-of-pocket payments versus the payment of health insurance premiums, for both patients and individuals in the community.

# Research Plan and Methods

The proposed study will involve quantitative analysis of primary quantitative data from household interviews and health facility exit interviews.

## Primary Data Collection and Analysis

### Study Sites

Primary data collection will take place in the Kilombero district in the Morogoro region, and Same district in the Kilimanjaro region. Both regions are primarily rural with a town center at Ifakara and Same town, respectively. Kilombero District was chosen due to the long-standing research collaborations with IHI in Kilombero as Ifakara is the main field center. Same district was selected because of the similar health care infrastructure including number of hospitals, health facilities, and dispensaries, and similar rural/urban mix as Kilombero, but with differing health policies as it is located in a different region. The study will take place at both healthcare facilities and in private households throughout the two districts.

### Study Design

This study will use primary data that will be collected in two surveys: A general household survey including biometric testing for hypertension and diabetes, and an exit questionnaire administered to patients following their visit at a health facility.

### Sampling Strategy and Sample Size

#### *Household survey*

We will conduct a household survey to collect information on household demographic and economic characteristics, health insurance and social protection, chronic illness, healthcare access and utilisation, household health expenditure, out of pocket payments for health care, and coping strategies to deal with diseases. In order to answer the key research question, information will be collected regarding direct and indirect household out-of-pocket expenditure (OOPs) on healthcare, how the household pays for their OOPs or social health protection premiums, economic productivity loss due to illness, recent healthcare utilisation, and the role of one’s social network in coping with illness. For respondents that accept, we will also measure blood pressure and random blood sugar to quantify undiagnosed and untreated hypertension and possible diabetes in the population. We will counsel patients with results outside of the normal range of the importance of following up with a healthcare provider to formally diagnose and initiate treatment if needed.

Using a stratified sampling approach, a household survey will be delivered throughout Kilombero and Same districts. In order to detect associations with the primary outcome of household health expenditure (power of 0.80, significance level of 0.05), a minimum sample size of 202 households per district is required. To calculate the minimum sample size, a household health expenditure of 30,000 Tanzanian shillings was used as a benchmark; corresponding to the cost of enrolling a household in the iCHF. However, in order to also answer research questions associated with our secondary objectives, we will seek a total sample size of 390 households per district.

As such, in each of Kilombero and Same district, 15 households from each district’s only town (Ifakara and Same, respectively), and 15 households from a random selection of 25 other villages will be sampled (Table 1).

#### *Exit survey*

The patient health facility exit survey will be administered to patients at all tertiary and secondary health facilities, and a corresponding sample of 8 dispensaries in both the Kilombero district and Same district. As each of these districts has 8 health centres, dispensary sampling will be matched by randomly selecting 1 dispensary from each ward containing a health centre.

The patient exit survey will be delivered at health facilities to adult patients seeking outpatient care. Target sample size was designed to be representative of patents visiting the health facilities for outpatient care for all conditions included NCDs. Hospital and health centre outpatient clinics typically designate one day per week as an ‘NCD day’, when a medical officer is assigned to be available to provide outpatient NCD care. As such, for each sampled hospital and health centre, the exit surveys will be delivered on one health facility NCD day to ensure that sufficient numbers of patients with NCDs can receive the questionnaire, and on one clinic day not designated for NCD care so as to also collect a more typical sample of people seeking outpatient care. Some rural health facilities, especially dispensaries which might not offer an NCD clinic day, might have low numbers of patients specifically visiting for chronic disease care, but this is representative of the typical clinical makeup and should be measured.

Rather than random or systematic random sampling, this study will use the more operationally efficient method of selecting and recruiting patients based on the order in which they enter the consultation room. As demonstrated by Geldseltzer et al. (2016), sampling patients as they enter the consultation room is advantageous as it is more efficient and simple to implement than random sampling approaches, and it minimises the bias of consultation length that is associated with sampling patients as the exit the consultation room [10].

It is expected that with 2 field workers per health facility, 36 patients can be interviewed per day at hospitals and health centres, while 1 one field worker is expected to be able to interview 12 patients per day at each sampled dispensary. If patient volume is too low to meet this daily requirement, field workers will return later to the same facility to ensure the sample size is reached. As such, across both districts, we are anticipating a total sample size of 1,632 for the exit survey (Table 1), generously exceeding the minimum sample size that was previously indicated. Participants will be recruited while waiting to enter the consultation room, and the research assistant will administer the questionnaire after the participants has seen the clinician so as to avoid disrupting the work flow at the clinic.

The questions asked in the exit survey will mostly be the same as those asked in the household survey, so that household survey respondents will serve as the general population with which people accessing NCD care can be compared. However, in the exit surveys, additional questionnaire items will be included relating to the specific services being utilised on the day of the health facility visit. The questionnaire will take on average 20 minutes to complete. If during the piloting phase the questionnaire takes too long to complete, we will reduce the number of questions asked from the accepted survey, prioritizing questions that best reflect the explicit research questions.

**Table 1: Sample size and sampling strata.** Sample size calculated based on a hypertension prevalence of 19.9% (95% CI: 17.1-22.9) for rural Tanzania [11], population estimates from the Tanzania 2012 census, and a benchmark mean annual household health expenditure of 30,000 Tanzanian shillings by using a modified Cochran sample size calculation.

| **Study Type** | **Sample Size** | **Strata** |
| --- | --- | --- |
| Exit Interviews |  | Kilombero District:   - 18 health facilities |
|  | 72 per hospital | - 2 hospitals |
|  | 72 per health centre | - 8 health centres |
|  | 12 per dispensary | - 8 dispensaries |
|  |  | Same District:   - 18 health facilities |
|  | 72 per hospital | - 2 hospitals |
|  | 72 per health centre | - 8 health centres |
|  | 12 per dispensary | - 8 dispensaries |
|  | **Total: 1,632** | **Total: 36 health facilities** |
| Household Survey |  | Kilombero District: |
|  | 15 households | Ifakara town |
|  | 15 households per village | Sample of 25 villages |
|  |  | Same District: |
|  | 15 households | Same town |
|  | 15 households per village | Sample of 25 villages |
|  | **Total: 790 households** | **Total: 52 towns/villages** |

### Data Management

All data collected will be coded and be kept strictly confidential. All names will be replaced by unique identifying codes, and the coding matrix will be stored securely and separate from the data itself. All data will be collected and saved on electronic supports and stored on secure servers at the home institutions in Tanzania. The core research team is composed of team members located at IHI and Swiss TPH with different specializations. Data analysis will be done in collaboration, where all the researchers will have access and work on the data as per all collaborative and data management requirement. Any data transfer will take place either over secured networks or in person to a member of the research team. Data will be used for the sole purposes of the research project and published data will not include any kind of information that can be linked to individuals.

### Analytical Methods

By delivering a household survey to the general populations of Kilombero and Same, and an exit survey to people accessing NCD services, we will be able to comparatively analyse the specific challenges and coping mechanisms that people living with NCDs face in accessing services, in relation to the general population.

The data collected from the household survey and exit survey will first be analysed using descriptive statistics to investigate the burden of OOPs and economic losses associated with living with hypertension and/or diabetes, and to determine how much households spend upon the direct and indirect costs of accessing NCD care. General hypotheses related to the geographic and economic accessibility of health care, such as whether distance from a health facility is associated with direct and indirect healthcare costs, and whether distance from a health facility moderates the association between social health protection and household health expenditure, will be explored using linear mixed models and analyses of variance.

We will quantify the proportion of the household survey respondents who were hypertensive or diabetic, and not aware of their condition or not enrolled in care. We will then use univariate and multivariate logistic regression analyses to identify differences between the household respondents with an NCD who are not enrolled in care and NCD patients from the exit interviews who are enrolled in care. We will quantify systematic and individual characteristic predictors of willingness-to-pay for NCD care and social health protection schemes for people in care for non-NCD reasons, for NCD reasons, and household respondents.

A regression model will then be used to assess whether living with hypertension and/or diabetes is associated with financial hardship, and/or illness-related economic and productivity losses, using the indicators catastrophic health expenditure and impoverishment. An analysis of variance (ANOVA) of mean spending by revenue source will then be used to determine the relative importance of financial contributions from various household revenue streams to covering the costs of NCD care, such as:

- Earnings from work (informal or formal)

- Selling personal property (clothing, bicycle, furniture, etc.)

- Financial contributions from the social network (friends, family, neighbours, etc.)

- Other means of support from the social network, such as accompaniment to the health facility, or offering transport to the health facility

- Social health protection, or other forms of social protection (CHF, NHIF, TASAF, etc.)

Egocentric social network analysis will then be used to analyse and visualise the network of actors and individuals that support people living with NCDs, and to characterise and quantify the function of a patient’s support network. Similar in concept to the more typical sociocentric social network analysis, egocentric social network analysis instead will analyse the composition and function of a network of actors in relation to a single entity; in this case, an individual with hypertension and/or diabetes). The main units of the egocentric social network analysis will be the composition of the patient’s support network (type of relationship, propinquity, age, gender, etc.), and the type of social support that is being received and provided (cash, personal care, clothing, etc.). The questionnaire instrument relevant to the proposed study has been included in this IRB submission package.

# Research Ethics

Ethical clearance for this study will be required from the Ethikkommission Nordwest- und Zentralschweiz (EKNZ), the Institutional Review Board of Ifakara Health Institute (IHI), the Tanzanian National Institute for Medical Research (NIMR), the Tanzania Commission for Science and Technology (COSTECH).

Informed consent will be obtained from all those participating in the surveys. All participants will be informed about the objectives of the research and that they are free to choose to participate or not, that the research will involve minimal risks, and that they may choose to participate or withdraw from the study with no adverse consequences arising from their decision. In Tanzania, district authorities will be contacted to seek permission to interview individuals within the administration. Health facility administrators will be contacted to seek permission to conduct exit interviews with patients at their facilities. All data will be treated as confidential and presented publicly only in aggregate or anonymized form. No personal identifying details of any study participant will be linked with the information provided by them.

# Dissemination Plan

Firstly, the findings of the proposed studies will be presented and reported to stakeholders in Tanzania by organising meetings and presentations with representatives and officials of the Kilombero and Same district, the NHIF, iCHF, the Ministry of Health, PoRALG, and other interested stakeholders. Findings will be subsequently presented at academic conferences, and published in open-access, peer-reviewed scientific journals. It is expected that findings from the research involving secondary data analysis will be available to be presented at the 2021 Congress of the International Health Economics Associated (iHEA), to be hosted by the University of Cape Town in July 2021. A list of scientific articles that will be tentatively produced during the course of the proposed study is provided in table 2.

# List of Investigators and their Roles

Sally Mtenga- Principal Investigator

Fabrizio Tediosi- Principal Investigator

Brady Hooley- Investigator, PhD student assigned role of supervising field work

Brianna Osetinsky- Investigator, postdoctoral scientist assigned role of supervising field work

Kassimu Tani- Investigator, PhD student assigned role of supervising field work

Grace Mhalu- Investigator

# References

1. Jakovljevic M, Jakab M, Gerdtham U, McDaid D, Ogura S, Varavikova E, et al. Comparative financing analysis and political economy of noncommunicable diseases. J Med Econ. 2019;22: 722–727. doi:10.1080/13696998.2019.1600523

2. Bintabara D, Mpondo BCT. Preparedness of lower-level health facilities and the associated factors for the outpatient primary care of hypertension: Evidence from Tanzanian national survey. PLOS ONE. 2018;13: e0192942. doi:10.1371/journal.pone.0192942

3. Peck R, Mghamba J, Vanobberghen F, Kavishe B, Rugarabamu V, Smeeth L, et al. Preparedness of Tanzanian health facilities for outpatient primary care of hypertension and diabetes: a cross-sectional survey. Lancet Glob Health. 2014;2: e285–e292. doi:10.1016/S2214-109X(14)70033-6

4. Siddharthan T, Ramaiya K, Yonga G, Mutungi GN, Rabin TL, List JM, et al. Noncommunicable Diseases In East Africa: Assessing The Gaps In Care And Identifying Opportunities For Improvement. Health Aff Proj Hope. 2015;34: 1506–1513. doi:10.1377/hlthaff.2015.0382

5. Boerma T, Eozenou P, Evans D, Evans T, Kieny M-P, Wagstaff A. Monitoring Progress towards Universal Health Coverage at Country and Global Levels. PLOS Med. 2014;11: e1001731. doi:10.1371/journal.pmed.1001731

6. Borghi J, Mtei G, Ally M. Modelling the implications of moving towards universal coverage in Tanzania. Health Policy Plan. 2012;27: i88–i100. doi:10.1093/heapol/czs009

7. Jan S, Laba T-L, Essue BM, Gheorghe A, Muhunthan J, Engelgau M, et al. Action to address the household economic burden of non-communicable diseases. Lancet Lond Engl. 2018;391: 2047–2058. doi:10.1016/S0140-6736(18)30323-4

8. Evans IEM, Martyr A, Collins R, Brayne C, Clare L. Social Isolation and Cognitive Function in Later Life: A Systematic Review and Meta-Analysis. J Alzheimers Dis JAD. 2019;70: S119–S144. doi:10.3233/JAD-180501

9. Jaspers L, Colpani V, Chaker L, van der Lee SJ, Muka T, Imo D, et al. The global impact of non-communicable diseases on households and impoverishment: a systematic review. Eur J Epidemiol. 2015;30: 163–188. doi:10.1007/s10654-014-9983-3

10. Geldsetzer P, Fink G, Vaikath M, Bärnighausen T. Sampling for Patient Exit Interviews: Assessment of Methods Using Mathematical Derivation and Computer Simulations. Health Serv Res. 2018;53: 256–272. doi:10.1111/1475-6773.12611

11. Ploth DW, Mbwambo JK, Fonner VA, Horowitz B, Zager P, Schrader R, et al. Prevalence of CKD, Diabetes, and Hypertension in Rural Tanzania. Kidney Int Rep. 2018;3: 905–915. doi:10.1016/j.ekir.2018.04.006

# Annex I

## Tables of requested variables NHIF

| Variable from NHIF Claims | Type | Category Breakdown |
| --- | --- | --- |
| Anonymized Identification Number | Numerical |  |
| Birthdate | Date |  |
| Gender | Category | Male, Female |
| Claim value | Numerical |  |
| Treatment date | Date |  |
| Facility Name | String |  |
| Facility Ownership | Category | Faith Based, government, non-government, private |
| Facility Level | Category | Accredited drug dispensing, Dispensary, District hospital, health centre, national referral hospital, pharmacy, regional referral hospital, specialized clinics, laboratories and diagnostic, zone referral hospital |
| Ward | Category |  |
| District | Category |  |
| Region | Category |  |
| Services Type | Category | Medicine and Consumables, Diagnostic Examinations |
| Service Detail | String |  |
| Funeral/Death Expenses | Numerical | Will be used to assess mortality |
| Life Insurance payout | Numerical | Will be used to assess mortality |

| Variable from NHIF Beneficiaries Records | Type | Category Breakdown |
| --- | --- | --- |
| Anonymized Identification Number | Numerical |  |
| Relationship To Policy Holder | Category | Principal, spouse, parent, child, other |
| Member Category | Category | Askari, Clerics, Councillor, Public Employee, Member of Parliament, Mutual-Principal, Private Employee, Private Individual,Retiree, Student and interns, toto afya kadi, KFW |
| Contribution | Numerical |  |
| Wealth Quintiles | Categorical drawn from numerical |  |
| Birthdate | Date |  |
| Gender | Category | Male, Female |
| Member enrollment Date | Date |  |
