# Supplementary material for: Care cascades for hypertension and diabetes: Cross-sectional evaluation of rural districts in Tanzania
Source: PLoS Med. 2022 Dec 5;19(12):e1004140. doi: 10.1371/journal.pmed.1004140 (PMC9762578; doi:10.1371/journal.pmed.1004140)
Supplement: S1 Table — (DOCX) [file pmed.1004140.s004.docx]

**Table A: Population Adjusted Prevalance and Care Cascade for Hypertension and Diabetes**

| Using Survey Weighting | N | Hypertension | N | Diabetes |
| --- | --- | --- | --- | --- |
| Positive for Condition | 773 | 24.1  (20.4 - 27.8) | 707 | 2.09  (1.34 - 2.84) |
| Previously Diagnosed with Condition | 316 | 37.64  (31.21 - 44.06) | 42 | 86.79  (75.03 - 98.55) |
| Engaged in care | 316 | 19.99  (15.36 - 24.62) | 42 | 74.99  (59.26 - 90.72) |
| Retained in Care | 316 | 16.64  (12.29 - 20.98) | 42 | 74.99  (59.26 - 90.72) |
| Condition Controlled | 316 | 7.64  (4.50 - 10.77) | 42 | 56.03  (38.42 - 73.64) |

S1 Table presents the prevalance and care cascade steps adjusted for the age and gender demographic structure of the underlying tanzanaina population drawn from the 2020 estimates for Tanzania from the United Nations World Population Prospects.
