# Supplementary material for: Care cascades for hypertension and diabetes: Cross-sectional evaluation of rural districts in Tanzania
Source: PLoS Med. 2022 Dec 5;19(12):e1004140. doi: 10.1371/journal.pmed.1004140 (PMC9762578; doi:10.1371/journal.pmed.1004140)
Supplement: S2 Table — (DOCX) [file pmed.1004140.s005.docx]

**S2 Table: Care Cascade Number of Events in Each stage of the care cascade**

| Characteristic | Previously Diagnosed | Engaged in care | retained Treated | Controlled |
| --- | --- | --- | --- | --- |
| Kilombero | 61 | 33 | 24 | 12 |
| Same | 77 | 49 | 42 | 24 |
| Female | 98 | 53 | 45 | 26 |
| Male | 40 | 29 | 21 | 10 |
| Mean Age (Years) | 54 | 59 | 57 | 56 |
| Urban/Peri-Urban | 50 | 30 | 25 | 13 |
| Rural | 88 | 52 | 41 | 23 |
| Marital Status |  |  |  |  |
| Married/Living Together | 96 | 58 | 48 | 26 |
| Divorced/Widowed/Single | 42 | 24 | 18 | 10 |
| Highest Level Education Completed |  |  |  |  |
| Did Not Complete Any School | 29 | 16 | 10 | 6 |
| Primary School | 94 | 56 | 48 | 25 |
| Secondary School or higher | 15 | 10 | 8 | 5 |
| Occupation |  |  |  |  |
| Formal Sector  (Civil Servant / Private Formal) | 1 | 1 | 1 | 1 |
| Farming | 91 | 53 | 44 | 24 |
| Self Employed/Small Business | 22 | 10 | 10 | 6 |
| Care For Home/Children | 5 | 4 | 3 | 2 |
| Retired | 19 | 14 | 8 | 3 |
| Social Health Protection |  |  |  |  |
| No Social Health Protection | 89 | 47 | 36 | 22 |
| iCHF Health Insurance | 3 | 3 | 3 | 2 |
| NHIF Health Insurance | 23 | 16 | 16 | 6 |
| Other private insurance | 4 | 2 | 2 | 1 |
| Health Care Fees Exemption | 19 | 14 | 9 | 5 |
| Diagnosis of Comorbid Diabetes | 18 | 15 | 10 | 5 |

This presents the number of observations for the categorical variables and the mean for the continuous for the independent variables of in all four stages of the care cascade.
