# Supplementary material for: Care cascades for hypertension and diabetes: Cross-sectional evaluation of rural districts in Tanzania
Source: PLoS Med. 2022 Dec 5;19(12):e1004140. doi: 10.1371/journal.pmed.1004140 (PMC9762578; doi:10.1371/journal.pmed.1004140)
Supplement: S3 Table — (DOCX) [file pmed.1004140.s006.docx]

**S3 Table: Reasons why medication was not obtained at the last healthcare visit**

| Reason why medication for chronic disease was not obtained | Frequency | % |
| --- | --- | --- |
| Health facility pharmacy was out of stock | 33 | 84.62 |
| I could not afford the medicines | 3 | 7.69 |
| Other | 3 | 7.69 |

Respondents that were engaged in care were asked if they obtained prescribed medications on their last healthcare visit for their chronic condition, and those that responded in the negative were asked why. The responses are presented here
