# Supplementary material for: Care cascades for hypertension and diabetes: Cross-sectional evaluation of rural districts in Tanzania
Source: PLoS Med. 2022 Dec 5;19(12):e1004140. doi: 10.1371/journal.pmed.1004140 (PMC9762578; doi:10.1371/journal.pmed.1004140)
Supplement: S4 Table — (DOCX) [file pmed.1004140.s007.docx]

S4 Table presents the sensitivity analysis for the definition of engagement in care. We varied the definition of engaged in care from very restricted at a monthly follow up schedule for all (1 Month), a three month follow up schedule for everyone regardless of measured blood pressure at the time of the survey (3 Month), and a six month follow up schedule at the time of our survey (6 Month).

Compared to the definition used in the main analysis, the most restricted follow up on monthly basis resulted in a decrease for people living in rural settings, an increase in odds the respondents registered with the NHIF, and a loss of significance for differences in gender, educational attainment, health care fee exemption, and a diagnosis of comorbid diabetes. For the follow up schedule of every three months, the changes compared to the definition in the main analysis resulted in a loss of significant differences across gender, and a decrease in the magnitude of the odds for diagnosis of comorbid diabetes. For the most conservative estimation of all respondents having a six month follow up schedule, the only difference was the decrease in the odds of being engaged in care for the diagnosis of comorbid diabetes compared to the definition used in the main analysis.

**S4 Table: Sensitivity Analysis, regression results for the outcome Engagement in Care varying definition of Engaged in Care.**

| Characteristic | Primary Definition | 1 Month Follow Up | 3 Month Follow Up | 6 Month Follow Up |
| --- | --- | --- | --- | --- |
| Kilombero | REF | REF | REF | REF |
| Same | 1.20  [0.71,2.03] | 1.52  [0.82,2.82] | 1.08  [0.65,1.79] | 1.34  [0.81,2.22] |
| Female | REF | REF | REF | REF |
| Male | 0.51  [0.23,1.11] | 0.52  [0.22,1.22] | 0.55  [0.25,1.20] | 0.44^*^  [0.21,0.92] |
| Age | 1.04^*^  [1.01,1.07] | 1.04^*^  [1.00,1.07] | 1.04^**^  [1.01,1.07] | 1.04^**^  [1.01,1.07] |
| Urban/Peri-Urban | REF | REF | REF | REF |
| Rural | 0.69  [0.39,1.23] | 0.47^*^  [0.25,0.90] | 0.67  [0.39,1.15] | 0.65  [0.36,1.16] |
| Marital Status |  |  |  |  |
| Married/Living Together | REF | REF | REF | REF |
| Divorced/Widowed/Single | 0.48^*^  [0.25,0.93] | 0.60  [0.27,1.35] | 0.58  [0.29,1.18] | 0.49^*^  [0.26,0.94] |
| Highest Level Education Completed |  |  |  |  |
| Did Not Complete Any School | REF | REF | REF | REF |
| Primary School | 2.02  [0.98,4.18] | 1.64  [0.71,3.76] | 2.22^*^  [1.03,4.77] | 2.42^*^  [1.15,5.12] |
| Secondary School or higher | 2.17  [0.43,10.91] | 1.80  [0.29,11.36] | 3.03  [0.55,16.87] | 2.46  [0.52,11.56] |
| Occupation |  |  |  |  |
| Formal Sector  (Civil Servant / Private Formal) | REF | REF | REF | REF |
| Farming | 0.98  [0.06,16.29] | 0.88  [0.05,16.33] | 1.13  [0.06,20.04] | 1.29  [0.08,20.60] |
| Self Employed/Small Business | 1.04  (0.05 - 23.35) | 1.03  (0.04 - 27.12) | 1.13  (0.06 - 28.96) | 1.60  (0.08 - 30.60) |
| Care For Home/Children | 0.86  [0.04,17.17] | 0.88  [0.04,21.29] | 1.17  [0.06,24.83] | 1.30  [0.08,21.67] |
| Retired | 3.64  (0.16 - 91.14) | 2.30  (0.10 - 90.20) | 2.52  (0.10 - 62.14) | 2.34  (0.09 - 58.01) |
| Social Health Protection |  |  |  |  |
| No Social Health Protection | REF | REF | REF | REF |
| iCHF Health Insurance | 1.07  [0.21,5.36] | 0.48  [0.09,2.54] | 0.83  [0.18,3.77] | 1.04  [0.27,4.04] |
| NHIF Health Insurance | 1.92  (0.61 - 6.05) | 3.65**  (1.36 - 9.78) | 2.46  (1.22 - 6.92) | 2.10  (0.78 - 5.64) |
| Other private insurance | 2.00  [0.67,5.93] | 3.69^*^  [1.35,10.08] | 2.56  [0.92,7.11] | 2.15  [0.79,5.81] |
| Health Care Fees Exemption | 3.16**  (1.01 - 9.86) | 2.24  (0.72 - 6.88) | 3.51*  (1.23 - 9.99) | 4.19**  (1.64 - 10.74) |
| Diagnosis of Comorbid Diabetes | 0.68  [0.12,3.87] | 1.26  [0.25,6.40] | 0.91  [0.17,4.90] | 0.61  [0.12,3.20] |

Exponentiated coefficients; 95% confidence intervals in brackets, iCHF is improved Community Health Fund, NHIF is National Health Insurance Fund.

^*^ *p* < 0.05, ^**^ *p* < 0.01, ^***^ *p* < 0.001
