# Supplementary figures and images for: Care cascades for hypertension and diabetes: Cross-sectional evaluation of rural districts in Tanzania
Source: PLoS Med. 2022 Dec 5;19(12):e1004140. doi: 10.1371/journal.pmed.1004140 (PMC9762578; doi:10.1371/journal.pmed.1004140)

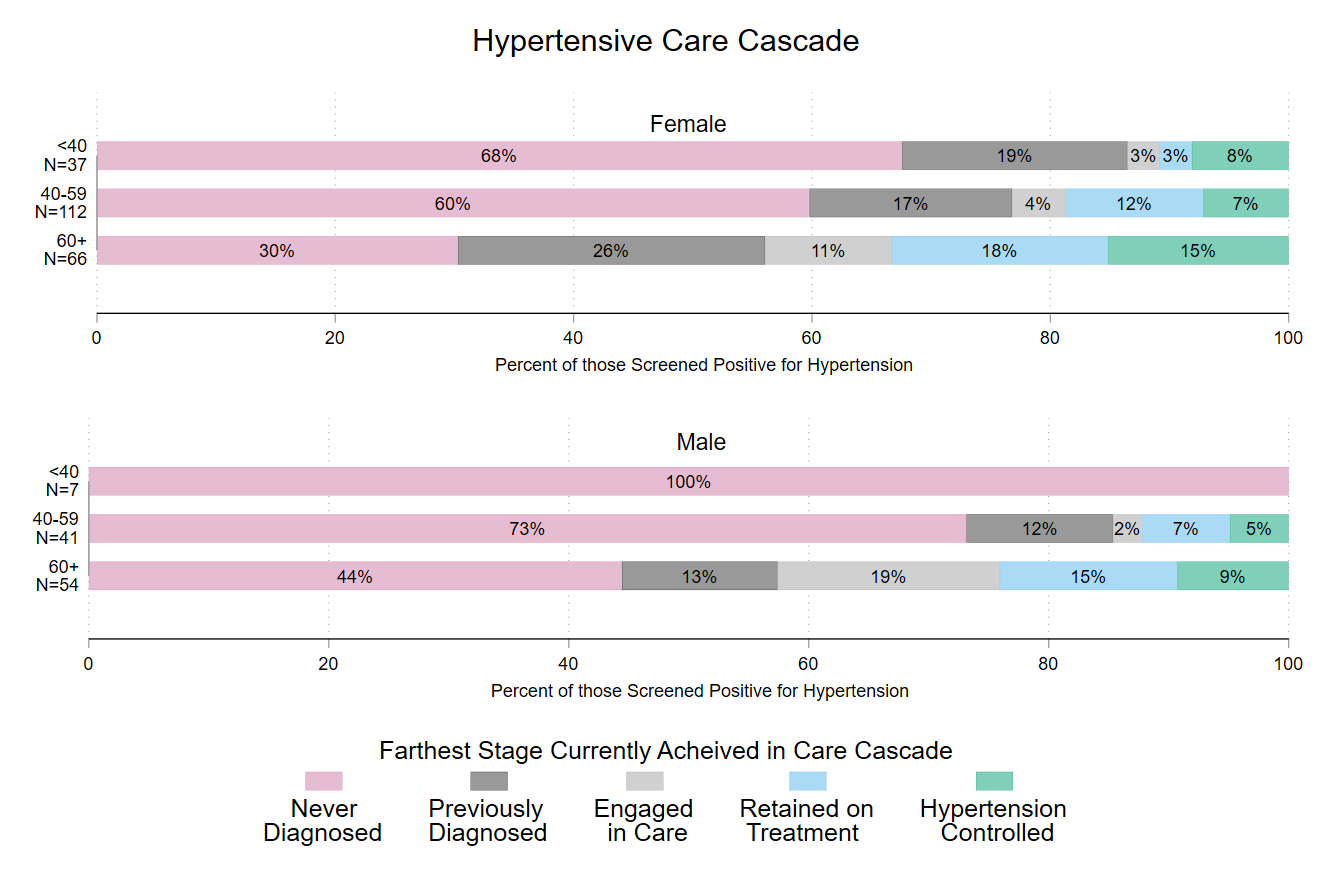

Supplement: S1 Fig — This shows the proportion of those who screened positive for hypertension in the farthest point along the hypertension care cascade at the point of measurement, disaggregated by age group and gender. (TIF) [file pmed.1004140.s008.tif]

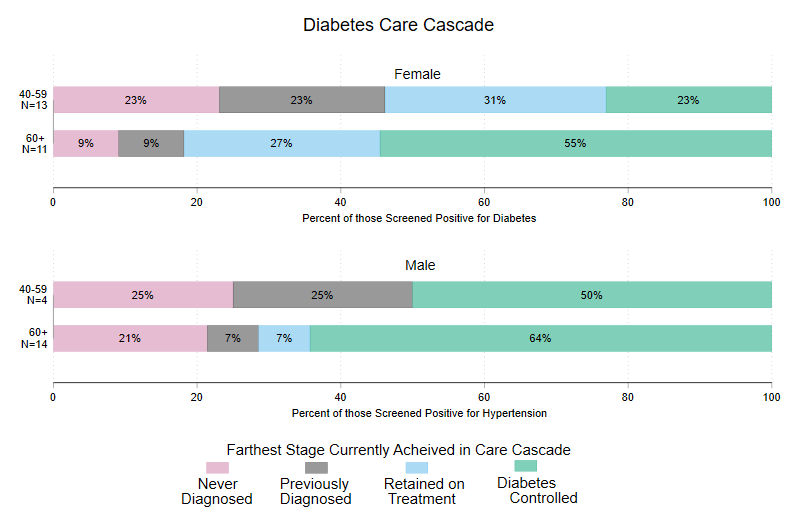

Supplement: S2 Fig — This shows the proportion of those who screened positive for diabetes in the farthest point along the diabetes care cascade at the point of measurement, disaggregated by age group and gender. (TIF) [file pmed.1004140.s009.tif]

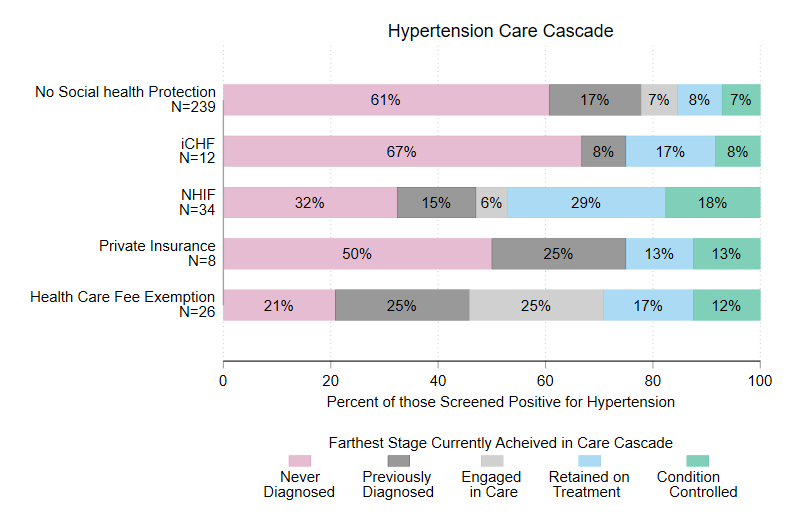

Supplement: S3 Fig — This shows the proportion of those who screened positive for hypertension in the farthest point along the hypertension care cascade at the point of measurement, disaggregated by social health protection status. (TIF) [file pmed.1004140.s010.tif]

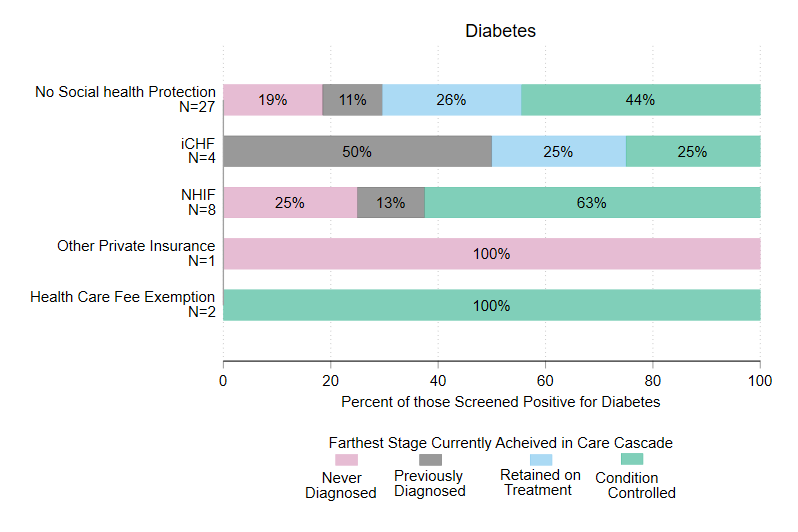

Supplement: S4 Fig — This shows the proportion of those who screened positive for diabetes in the farthest point along the diabetes care cascade at the point of measurement, disaggregated by social health protection status. (TIF) [file pmed.1004140.s011.tif]
